# Fatal drug-induced immune hemolytic anemia due to cefotetan; A case study

**DOI:** 10.4103/0973-6247.39507

**Published:** 2008-01

**Authors:** Jim Perkins

**Affiliations:** *Director, ENH Blood Banks, Evanston, IL, USA*

**Keywords:** Cefotetan, direct antiglobulin test, drug-induced immune hemolytic anemia

## Abstract

A case is described here of drug-induced immune hemolytic anemia (DIIHA) due to cefotetan administered to a post-partum woman who received the drug for infection prophylaxis at the time of caesarean section. Renewed fatal hemolysis occurred when the drug was given a second time 12 days after the first dose. The initial immunohematologic findings included a positive direct antiglobulin test (DAT) due to IgG and complement coating of the patient’s RBCs as well as an eluate that did not react with RBCs in the absence of drug. The antibody was drug-dependent, reacting with both drug-coated RBCs as well as when the drug was added to a mixture of her serum and donor RBCs. Cefotetan has been a common cause of this uncommon problem. The clinical features of cefotetan DIIHA, classification of drug-induced antibodies, and the differential diagnosis of a positive DAT are briefly discussed.

## Introduction

Drug-induced immune hemolytic anemia (DIIHA) is rare and difficult to diagnose, both in the clinic and in the immunohematology laboratory. Arndt and Garratty reported that cefotetan was the responsible drug in 72% of DIIHA cases referred to their laboratory between 1995 and 2004.[1] A similar retrospective review of the experience of another prominent reference laboratory reports that half of all drug-related antibodies they investigated were directed against cephalosporins, and of those, 73% were directed against cefotetan.[2] The manufacturer ceased production of cefotetan in 2005. Nonetheless, this case report is presented in the hope that it will help the reader familiarize with the laboratory features of DIIHA and increase the index of suspicion for the diagnosis of DIIHA.

## Case Report

A 30-year-old woman underwent caesarean section for her third pregnancy. Immediately before and after the operation she received doses of intravenous cefotetan for prophylaxis against bacterial infection. Prior to admission, she was taking cephalothin orally. There were two previous uncomplicated term pregnancies. She had never been transfused, and her routine prenatal blood group antibody detection test (antibody screen) was negative. She was discharged on the second post-operative day, apparently without complications. A complete blood count (CBC) before discharge documented a hemoglobin of 11.4 g/dL and hematocrit of 32.9.

On the 7^th^ day after delivery a nurse visiting her at home did not observe any obvious problems. The patient did well until 11 days after delivery when she felt faint. She reported this to her physician who thought she might have hypoglycemia. The following day she noted vaginal bleeding, fatigue, malaise and jaundice and visited her physician. A CBC revealed a hemoglobin of 5.1 g/dL, hematocrit 14.6, white blood cells (WBCs) 22,000 and platelets 524,000. She was admitted to the hospital for transfusion at noon.

Two units of red blood cells (RBCs) were ordered. Pre-transfusion testing [Table 1] demonstrated negative antibody detection test and cross-matches, but the autocontrol was 2+ reactive in the anti-human globulin (AHG) phase of testing [Tables 1, 2]. Transfusion was delayed due to a concern over the possibility of finding compatible RBCs [Table 3]. Further work-up included a positive direct antiglobulin test (DAT) with poly-specific anti-human globulin and anti-IgG [Table 4]. An eluate prepared from her RBCs was non-reactive. These findings were confirmed by a reference laboratory towards the evening and prior to transfusion. Other laboratory data included a normal total bilirubin (1.4 mg/dL, upper limit of normal 1.5) and a normal LDH level. A sonogram showed a hematoma anterior to the uterus. Oral ampicillin was administered at 16:00 and 20:00 for low-grade fever.

**Table 1:** ABO and Rh typing

Specimen	Anti-			Test cells		Anti-		Interpretation	
				
	A	B	AB	A1	B	D	Control	ABO	Rh
Initial	4+	0	4+	0	4+	4+	0	A	Pos
Post-cephotetan	4+	0	4+	0	4+	4+	0	A	Pos

**Table 2:** Antibody detection test

	Initial specimen				Eluate		Post-cefotetan specimen			
			
	IS*	30′37°	AHG†	CCC‡	AHG	CCC	IS	30′37°	AHG	CCC
SCI**	0	0	0	2+	0	2+	0	0	4+	
SCII	0	0	0	2+	0	2+	0	0	4+	
SCIII	0	0	0	2+	0	2+	0	0	4+	
Auto††	0	0	2+				0	0	4+	

* IS, immediate spin (phase of testing)

† AHG, anti-human globulin

‡ CCC, Coombs' control cells

** SC followed by a roman numeral indicates an antibody screening cell

†† Auto; autocontrol

**Table 3:** Cross-matches

Unit	Initial specimen				Post-cefotetan specimen			
		
	IS	30′37°	AHG	CC	IS	30′37°	AHG	CC
1	0	0	0	2+	0	0	4+	
2	0	0	0	2+	0	0	4+	
3	0	0	0	2+	0	0	4+	
4	0	0	0	2+	0	0	4+	
5	0	0	0	2+	0	0	4+	
6	0	0	0	2+	0	0	4+	

**Table 4:** Direct antiglobulin test

Specimen	POLY*	Anti-IgG	Anti-C3
Initial	2+	2+	NT
Post-cefotetan	NT†	3+	NT

* POLY, polyspecific anti-human globulin

† NT, not tested

After discussion with the blood bank, a pathologist and the attending physician, RBCs were made available. At approximately 22:30, the temperature had increased to 102.2°F, and 2 g of cefotetan was administered intravenously. At 23:00, transfusion was started with an ‘*in vivo* cross-match’ with 50 mL of RBCs. Soon thereafter, the patient was restless and anxious with nausea, vomiting and complained of abdominal pain. Transfusion was stopped and diphenhydramine was administered. The ‘*in vivo* cross-match sample’ did not show hemoglobinemia, although the DAT had increased in strength from 2+ to 3+, and the urine specimen was noted to contain ‘blood’ [Table 4]. Nonetheless, transfusion of the entire unit was initiated. A CBC at 23:30 revealed a hemoglobin of 4.2 g/dL and hematocrit 11.8.

The patient was transferred to the ICU. Her blood pressure was unstable. Transfusion was completed at 01:00, but the patient was lethargic and was oozing blood. A 2^nd^ unit was started, but was stopped after administering 50 cc when multiple blood samples exhibited hemolysis. A 3^rd^ unit of RBCs was started at 01:45, but stopped after 15 min. A CBC at 01:50 demonstrated a hemoglobin of 3 g/dL and hematocrit 7.6. Vital signs included pulse rates of 150-160, respiratory rates of 30-40 and blood pressures of 130-150/50-60. Repeat cross-matches of all the units with the original specimen were negative, but cross-matches with blood specimens drawn at 01:15 and 01:50 reacted 4+ with all RBCs tested. At this time, the DAT was 4+ with anti-IgG and very weakly positive (vw+) with complement, and the antibody detection test was 4+ positive with all cells as well. At 03:25 her hemoglobin was 1.8 g/dL and hematocrit 3.5. An elevated level of fibrin degradation products (>40, normal < 10) was noted. The patient died at 04:36, 16 h after entering the hospital.

Immunohematologic testing was performed both at the hospital and at the local blood center by standard methods.[3,4]

*Tests performed at the hospital:* Test results shown in Tables 1-4 are as outlined in the case report.

*Tests performed at the blood center reference laboratory:* Tests performed at the blood center confirmed the above results as shown in Tables 5 and 6.

**Table 5:** Repeat pre-transfusion testing, blood center

	Initial sample	Post-cefotetan
ABO	A	A
Rh phenotype	DEce	DEce, mixed field
DAT		
IgG	2+	4+
C3	w+	vw+
Antibody screen	Negative	3+*

* Positive at AHG phase with all cells

**Table 6:** Drug studies: initial sample

	Patient serum			Eluate		
		
	IS	60′37°	AHG	IS	60′37°	AHG
Cefotetan coated RBCs	4+		4+	0		4+
Control RBCs	0		0	0		0
Cefotetan + RBCs		3+, H*	3+		0	3+
Cefotetan + RBCs + C′†		4+, H	4+		0	3+
PBS‡ + RBCs		0	0		0	0
PBS + RBCs + C′		0	0		0	0
		**Controls without serum or eluate**				
Cefotetan + RBCs		0	0			
Cefotetan + RBCs + C′		0	0			

* H, hemolysis

† C, fresh serum as a complement source

‡ PBS, phosphate buffered saline

The testing for a drug-related antibody was performed but was not completed until after the patient died [Table 6].

## Discussion

This tragic case illustrates the clinical and immunohematologic features of severe cefotetan-related immune hemolytic anemia. This drug was often used for peri-operative prophylaxis against infection, particularly for caesarean section and gynecologic procedures. The immunohematologic test results will be discussed first followed by the clinical features of the case.

The initial tests performed at the hospital showed unremarkable results except for the positive auto-control. Since this test is performed by adding patient serum to a suspension of patient RBCs, incubating and then washing the RBCs, and then adding anti-human globulin, it is generally positive whenever the DAT is positive. A positive DAT was confirmed by the hospital laboratory, which demonstrated IgG coating the RBCs and by the blood center reference laboratory which showed that complement was coating the RBCs as well. In one report of 43 cases of anti-cefotetan, all patients had IgG on their RBCs and 37 (86%) had complement binding as well.[5] Many laboratories have stopped performing an auto-control in routine pre-transfusion testing. Ironically, had this test not been performed the laboratory would not have suspected a compatibility problem, and the patient might simply have been transfused early in her course without incident.

The drug study performed at the blood center reference laboratory demonstrated cefotetan-dependent RBC antibodies in the patient’s serum that reacted with drug-coated, washed RBCs as well as with uncoated RBCs in the presence of free drug. The former reactions are characteristic of penicillin-type drug-dependent antibodies (DDAs) or the so-called ‘drug adsorption’ mechanism in which the drug binds covalently with the elements on the RBC surface.[4] The drug-coated RBCs are then washed and reacted with patient serum to demonstrate DDA specificity. The reactions in the presence of free cefotetan are characteristic of quinine-type DDAs. The latter has been ascribed to an ‘immune complex’ mechanism, but the existence of immune complexes binding to the RBC via red cell Fc receptors has not been proven.[4] Most anti-cefotetan sera react in both formats, but many react with RBCs also in the absence of added or bound drug (‘auto-antibody’ mechanism).[5]

Johnson and coworkers have proposed a classification of drug-related antibodies that is based simply on the observed phenomena and does not use terms that presume a specific mechanism. Thus, drug-related antibodies are classified as drug-independent and -dependent. Within the drug-dependent category, they are sub-classified as “…either reacting with drug-treated RBCs (DTRC) or reacting in the presence of drug (IPOD).”

The blood group antibody detection test and all cross-matches were negative on admission but became positive after she received the last dose of cefotetan. This likely occurred because there was now drug in the patient’s serum to mediate antibody binding in these *in vitro* tests. Demonstrations of drug-independent reactions are most plausible if it can be proved that there is no drug in the serum being tested, for example, by first dialyzing the serum.

The patient appeared well on the 7^th^ post-partum, post-cefotetan day, first noting lightheadedness on the 11^th^ day. Since cefotetan has a half-life in the serum measured in hours, her initial hemolytic episode was presumably mediated by the drug bound to the RBCs and preserved in circulation in that state. In fact, it has been demonstrated directly that cefotetan can persist on the RBCs of treated patients for 16-92 days after administration.[6] The time course of DIIHA in this case and other cases reported as due to cephotetan[1] was unlike that caused by taking other drugs. That is, DIIHA typically occurs while the patient is taking the drug and subsides when the drug is stopped. Cefotetan DIIHA has previously been reported after one or two doses of the drug with onset of hemolysis 1 to 2 weeks later.[5]

On admission, this patient’s bilirubin and LDH were normal, indicating that hemolysis had slowed. However, after cefotetan was again administered hemolysis became very rapid, and the immune hemolytic anemia caused hemoglobinuria and disseminated intravascular coagulation (DIC) as shown by diffuse bleeding and elevated fibrin degradation products.

This case illustrates the importance of appreciating the possible causes of a positive DAT. Had this differential diagnosis been considered on admission this fatality could have been avoided. A positive DAT is seen in many hemolytic transfusion reactions, but this patient had not yet been transfused prior to admission. Transfusion of incompatible plasma causes a positive DAT, as when group ‘O’ platelets are given to a group ‘A’ recipient, but again, she had not been transfused. A positive DAT is characteristic of auto-immune hemolytic anemia, but if IgG auto-antibodies were coating the RBCs one would expect the eluate to have been reactive. A positive DAT with a negative eluate may be seen in patients with polyclonal hypergammaglobulinemia. Finally, these findings (positive DAT, negative eluate) are characteristic of drug-dependent antibodies when the eluate is reacted with antibody detection cells in the absence of the drug. If drug-dependent antibodies are suspected alternate drugs can be chosen. In this case, once cefotetan was administered on the second admission little could have been done to prevent her fatal hemolysis.
